# PyGMQL: scalable data extraction and analysis for heterogeneous genomic datasets

**DOI:** 10.1186/s12859-019-3159-9

**Published:** 2019-11-08

**Authors:** Luca Nanni, Pietro Pinoli, Arif Canakoglu, Stefano Ceri

**Affiliations:** Department of Electronics, Information and Bioengineering, Politecnico di Milano, Milan, Italy

**Keywords:** Genomic data, Data scalability, Tertiary data analysis, Distribution transparency, Python

## Abstract

**Background:**

With the growth of available sequenced datasets, analysis of heterogeneous processed data can answer increasingly relevant biological and clinical questions. Scientists are challenged in performing efficient and reproducible data extraction and analysis pipelines over heterogeneously processed datasets. Available software packages are suitable for analyzing experimental files from such datasets one by one, but do not scale to thousands of experiments. Moreover, they lack proper support for metadata manipulation.

**Results:**

We present PyGMQL, a novel software for the manipulation of region-based genomic files and their relative metadata, built on top of the GMQL genomic big data management system. PyGMQL provides a set of expressive functions for the manipulation of region data and their metadata that can scale to arbitrary clusters and implicitly apply to thousands of files, producing millions of regions. PyGMQL provides data interoperability, distribution transparency and query outsourcing. The PyGMQL package integrates scalable data extraction over the Apache Spark engine underlying the GMQL implementation with native Python support for interactive data analysis and visualization. It supports data interoperability, solving the impedance mismatch between executing set-oriented queries and programming in Python. PyGMQL provides distribution transparency (the ability to address a remote dataset) and query outsourcing (the ability to assign processing to a remote service) in an orthogonal way. Outsourced processing can address cloud-based installations of the GMQL engine.

**Conclusions:**

PyGMQL is an effective and innovative tool for supporting tertiary data extraction and analysis pipelines. We demonstrate the expressiveness and performance of PyGMQL through a sequence of biological data analysis scenarios of increasing complexity, which highlight reproducibility, expressive power and scalability.

## Background

By means of fast sequencing technologies, modern genomics promises to assist biological and clinical research by answering complex questions, e.g., how gene expression is deregulated in diseases, how mutations can lead to specific traits, how transcription factors interact to create complexes, how the genome organizes within three-dimensional configurations. To this aim, an impressive amount of sequencing data has been being collected by world-wide consortia as well as private laboratories and hospitals. Personalized medicine is slowly but steadily turning from vision into reality and biological research benefits more and more from bioinformatics approaches. In this scenario, computation approaches and tools are paramount to manage, process and analyze these large and heterogeneous collections of data.

Primary analysis can be defined as the machine specific steps needed to call base pairs and compute quality scores for those calls. As these steps generate what are referred to as “reads” of small nucleotide sequences, it’s left up to secondary analysis to reassemble these reads to get a representation of the underlying biology, as well as the detection of signals (primarily variants, but also expression levels and peaks of expression). Tertiary analysis focuses on the integration of these signals to answer research questions and diverges into a spectrum of various study specific downstream investigations [1].

Most of the effort of the bioinformatics and computational biology community was focused on primary and secondary analysis. Nowadays, the most important challenge is tertiary analysis, concerned with the development of complex models and tools for integrating and analyzing the heterogeneous pieces of information provided by secondary analysis, to the aim of producing novel biological knowledge. For addressing tertiary analysis, we proposed the Genomic Data Model (GDM) [2] and the GenoMetric Query Language (GMQL) [3, 4], composed by a query language and an engine built on top of Apache Spark [5]. GMQL enables heterogeneous dataset manipulation and provides a public repository of curated datasets to be used with private data but it is not suited for interactive data exploration or data analysis, as it assumes batch interaction from command lines or from a Web interface.

Tertiary data management practice requires the intertwining and seamless integration of data extraction and data analysis, so that the data scientist can easily build interactive applications which include use of statistical testing, machine learning and visualization. Python is earning more and more attention as vector language for data scientists. For this reason, we designed and implemented PyGMQL, a Python library which embeds the GMQL engine. PyGMQL combines the highly scalable approach of GMQL with the flexibility of Python, and solves the impedance mismatch between set-oriented execution of data management systems and the procedural nature of scripting languages. PyGMQL provides distribution transparency (the ability to address a remote dataset) and query outsourcing (the ability to assign processing to a remote service) in an orthogonal way. By relying on its Spark implementation, it can scale up from local execution to arbitrary cluster architectures. The ability to scale on parallel and cloud computing environments is the most innovative and distinguishing feature of PyGMQL and allows performing complex queries on large datasets. This, together with the high-level genomic operation definition and the possibility to embed complex data analysis workflows inside Jupyter Notebooks, makes PyGMQL a comprehensive tool for big genomic data exploration, management and integration.

### Related work

Several efforts have been done in the design of libraries or command line suites for genomic region manipulation. BEDTools [6] and BEDOPS [7] both offer Unix-based command line tools providing common BED file manipulation primitives. BEDTools also offers a Python interface [8]. In the R community, the GenomicRanges Bioconductor package [9] is a well-established tool with similar features. A more general purpose Python library for genomic data analysis is BioPython [10], which focuses more on secondary data analysis. These tools focus on supporting powerful operators for region manipulation upon a single experimental file. They emphasize usability but do not support scalable computing on remote clusters. Moreover, they do not include metadata and files must be individually loaded before being accessible to Python computations, which are performed by ad-hoc Python programs.

In [2] and [11] we provide respectively a functional and performance comparison of GMQL with BEDTools and BEDOPS, from which PyGMQL inherits the result.

PyGMQL has comparable expressive power in region manipulation with respect to these tools, but it adds an implicit iteration over all experiments of the same dataset. Its design is driven by scalability over thousands of experiments. Moreover, it supports metadata management explicitly, through high level operations for metadata extraction and for expressing predicates, that are seamlessly integrated with region manipulations. We designed the API of the library and its documentation so that biologists and bioinformaticians already competent in these tools can rapidly adapt to PyGMQL. For these reasons, PyGMQL can be considered as an upstream software for data manipulation both at the genomic data and metadata level, as demonstrated by several applications reported in the “Results” section. The results of PyGMQL computations can be easily then used by the previously cited tools.

The development of next-generation sequencing technologies has been followed by an increasing request of highly scalable software to define bioinformatics workflows [12]. This has brought to the development of very successful workflow management software like Galaxy [13], Snakemake [14], Nextflow [15] and FireCloud [16] (evolving to a new system named Terra, in May 2019). Most of the work done so far is tailored for secondary analysis pipelines like read alignment and mapping: important players in this field are the Genome Analysis Toolkit (GATK) [17] and the still in-development Hail framework^1^, both focusing on variant discovery. The ADAM software framework [18] is another important effort towards the deployment of common bioinformatics tools on big data management frameworks like Spark.

PyGMQL effectively complements these tools, by supporting data integration among heterogeneous data sources. We envision using PyGMQL at the end of secondary analysis pipelines, for supporting integrative analysis which also include access to large open repositories (as discussed in our application section). We already integrated both GMQL and PyGMQL with FireCloud and its evolution Terra^2^.

## Implementation

PyGMQL is part of a larger ecosystem of tools for biological data-driven research, that comprises a data manager (equipped with domain-specific data model and query language) and an open repository providing access to several public datasets as well giving users the possibility to import their private data; briefly reviewed next.

### Data model and query language

PyGMQL adopts the Genomic Data Model [3] to store and load genomic datasets. In GDM, a dataset consists of a set of samples, each observed on an individual or cell line in a given condition and typically represented as a track on the genome browser. Each sample includes two components: genomic regions (assignments from genomic coordinates, possibly stranded, to arbitrary genomic signals such as mutations, gene expressions, chip-seq peaks, topological domains, and so on) and arbitrary metadata (attribute-value pairs which describe the experimental/clinical/contextual conditions).

The GenoMetric Query Language [4] was developed to manipulate GDM datasets. It is inspired to relational algebra operators, which in turn have comparable expressive power as the SQL language. GMQL operators are either unary (UNOP - they apply to a single dataset) or binary (BINOP - they apply to two datasets) and produce one GDM dataset as result. The MATERIALIZE operator instructs the program where to store the result of a query. The request for materialization of a GMQL variable causes the recursive computation of all the intermediate datasets, up to the source datasets. Some of the operators are direct extensions of classic relational operations (i.e. SELECT, PROJECT, UNION, DIFFERENCE), while others target domain-specific region manipulations (i.e. COVER, MAP, JOIN). All operators in GMQL are applied both to genomic regions and their metadata; thus, it is possible to trace which samples of the input contribute to the samples of the result, as well as to compute global properties of the samples (e.g. statistics about their regions), using specific metadata attributes added during the computation while the query processes the datasets from the sources to the result.

### GMQL repository

Currently, the principal deployment of the GMQL system is through a Web application. For every user logged in the system, the GMQL repository reserves a private space to store his/her private data. In addition to this, the system collects a wide set of curated datasets from several sources which can be accessed by the users for integrative analysis. We integrated genomic metadata from five consolidated sources: The Cancer Genome Atlas [19] from Genomic Data Commons [20], ENCODE [21], Roadmap Epigenomics [22], and annotation data from GENCODE [23] and RefSeq [24]. We are in the process of adding other data sources, including Cistrome [25] for epigenomic and International Cancer Genome Consortium (ICGC, [26]) for mutation data, and we plan to integrate several other sources. GMQL offers several deployment settings for the repository, which can be installed on a local or Hadoop file system (HDFS).

### PyGMQL architecture

**Fig. 1:**
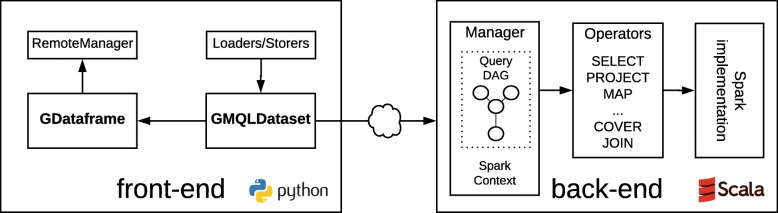
Schematic representation of the software components of PyGMQL. In the front-end, the GMQLDataset is a data structure associated with a query, referring directly to the DAG expressing the query operations. The GDataframe stores the query result and enables in-memory manipulation of the data. The front-end provides also a module for loading and storing data, and a RemoteManager module, used for message interchange between the package and an external GMQL service. The back-end interacts with the front-end through a Manager module, which maps the operations specified in Python with the GMQL operators implemented in Spark

PyGMQL is designed to address the specific needs of biologists and bioinformaticians during both the processes of pipeline design and data exploration [27]. PyGMQL offers a Python integrated environment where the users can interleave the definition of complex genomic queries and the analysis, manipulation and visualization of their results, which can then be stored and reused for further analysis or queries. The library adopts a client-server architecture, where the Python front-end exposes to the user all the dataset manipulation functions and utilities, while a Scala back-end implements all the query operators. As depicted in Fig. 1, the back-end relies on the implementation of GMQL on Spark.

PyGMQL offers a set of methods which wrap and extend the GMQL language operators Additional file 2. In Table 1 we show the mapping between the GMQL operators and their Python wrapper. Methods manipulate PyGMQL variables, each associated to structures called GMQLDataset. These keep a reference to an abstract GMQL representation, the GMQL directed acyclic graph (DAG) (see [4]), which represents the operations used for computing the variable. This design enables the back-end to apply query optimizations to the DAG structure [28]. PyGMQL adopts a *lazy execution* model, inspired by the Spark implementation. Therefore, no actual operation is executed until the materialize is applied to the variable.

**Table 1:** Mapping between PyGMQL methods and GMQL operators or utilities

PyGMQL function	Description	GMQL operator
load_from_path	UTIL, loads a dataset from local repository	SELECT
load_from_remote	UTIL, loads a dataset from remote repository	SELECT
load_from_file	UTIL, loads a bed file from local repository	
selectreg_selectmeta_select	UNOP, filters samples using region and/or metadata predicates	SELECT
projectreg_projectmeta_project	UNOP, projects (in/out) attributes of regions or metadata. Creates new attributes by means of expressions	PROJECT
extend	UNOP, creates a new metadata attribute by aggregation of region data	EXTEND
covernormal_coverflat_coversummit_coverhistogram_cover	UNOP, collapses regions from several samples into regions of a single sample, based on min/max accumulation indexes	COVER
order	UNOP, orders the samples of a dataset based on regions and/or metadata attributes	ORDER
merge	UNOP, merges all the samples of a dataset into a single one	MERGE
groupmeta_groupreg_group	UNOP, groups regions and/or metadata with the same values	GROUP
join	BINOP, joins the regions of two datasets based on distance-based predicates	JOIN
map	BINOP, computes aggregate values from overlapping regions of two datasets	MAP
union	BINOP, builds the union of regions and metadata of two datasets	UNION
difference	BINOP, keeps the regions of a dataset not intersecting with regions of another one	DIFFERENCE
materialize	UTIL, triggers the query execution for the specified dataset and stores the result after query completion	MATERIALIZE
head	UTIL, Shows the first lines of a dataset	

For every method we provide a concise explanation (UNOP stands for unary operator, BINOP stands for binary operator and UTIL identifies an utility function)

Once the query is terminated, its results are loaded in memory and stored in a GDataframe data structure, which holds both regions and metadata in the form of two Pandas DataFrames^3^. This makes it possible to work with the result of a query in the Python environment. It is also possible to convert the result of a query back to a GMQLDataset and use it as a new variable for a query. Obviously, the Python program can change its content before reloading.

To facilitate the integration with the Python ecosystem, PyGMQL enables to import datasets directly from Pandas DataFrames that use a BED or GTF format.

**Fig. 2:**
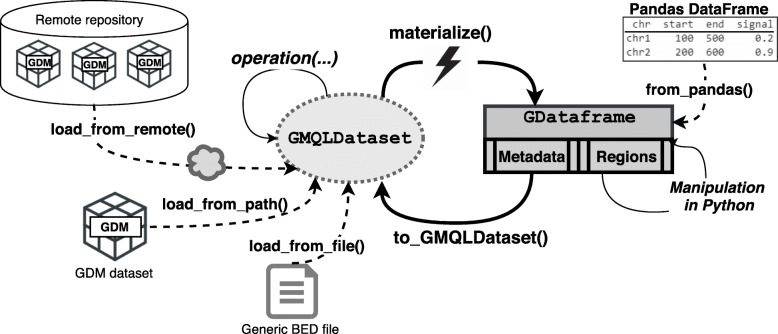
Relationships between GMQLDataset and GDataframe. Data can be imported into a GMQLDataset from a local GDM dataset with the load_from_path function. Using the load_from_file, it is possible to load generic BED files, while load_from_remote enables the loading of GDM datasets from an external GMQL repository, accessible through TCP connection. The user applies operation on the GMQLDataset and triggers the computation of the result with the materialize function. At the end of computation, the result is stored in-memory in a GDataframe, which can be then manipulated in Python. It is possible to import data directly from Pandas with from_pandas. Finally, it is possible to transform a GDataframe structure back into GMQLDataset using the to_GMQLDataset function

The interleaving between Python computation and GMQL execution constitutes a powerful tool for the bioinformatician, which is able to build complex pipelines without leaving the Python environment. By embedding PyGMQL within Jupyter Notebooks^4^, users can easily perform data exploration and are facilitated in reproducibility of their pipelines, a very important aspect of modern genomic computing. Figure 2 schematically represents the relationship between a GMQLDataset and a GDataframe, together with the main functions to load, materialize and import datasets from/to PyGMQL.

### Distribution transparency

The GMQL system is directly linked to one repository deployment, although many deployment technologies are supported. Instead, PyGMQL enables the users also to locally access their data, like most of the Python libraries. In addition, PyGMQL can also interface with an external GMQL system and login with the user credentials (using the login function) to interact with his/her private datasets or the public repository. During the query composition in PyGMQL, the user can specify if a source dataset comes from his local file system (using the load_from_path function) or from the remote GMQL repository (using the load_from_remote function). Therefore, queries in PyGMQL can be composed of genomic operations acting both on local and remote datasets.

**Fig. 3:**
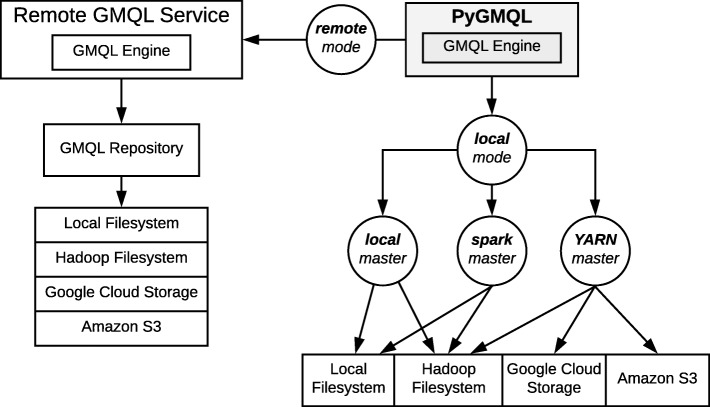
Deployment modes and executor options of the library. When the library is in remote mode, it interfaces with an external GMQL service, hosting a GMQL repository (accessible by the Python program, which has been deployed on several file systems). When the mode is set to local, the library can operate on various file systems, based on the selected master

Another important functionality of the library is given by its ability to "outsource" the query computation to an external GMQL service. If the users is logged on a remote GMQL server in PyGMQL, using the set_mode function she can decide if the query computation will be performed on the local (*local mode*) or on the remote (*remote mode*) system (Fig. 3). In the case of remote computation, the library takes care of downloading the result and loading it in a GDataframe.

The location of the datasets used during the query is orthogonal with respect to the mode of execution. Therefore, during the execution of a genomic query using both local and remote datasets, the library will manage their download or upload based on the mode. The library keeps tracks of the used datasets and their dependencies during the whole Python program execution, minimizing the data transmission between local and remote systems.

### Deployment over cloud infrastructures

When the mode of execution of PyGMQL is set to *local*, the user can specify the deployment strategy of the queries using the set_master function. Since the implementation of the GMQL operators is based on Spark, the library can be deployed in the following modes:

- *Local master*: the program is executed on the local user machine. In this execution mode, the PyGMQL software can access datasets in the local file system or in HDFS.
- *Spark master*: the back-end of the library is submitted to the master node of a Spark cluster and interacts with the front-end through a TCP connection. Also, in this case, the PyGMQL software can access datasets in the local file system (if the master node of the cluster is in the same machine as the python program) or in HDFS.
- *YARN master*: the back-end of the library is submitted to the Application Master of a YARN cluster and interacts with the front-end through a TCP connection. In this case, the local datasets reside on the Hadoop file-system. This deployment strategy must be adopted to run the library on cloud providers like Google Cloud and Amazon Web Services.

Figure 3 shows the available deployment modes and the possible master settings for the library together with the distributed file systems which the library can interact with.

## Results

We demonstrate the flexibility of the PyGMQL library through three data analysis workflows, available in the form of Jupyter Notebooks and scripts both in the Supplementary Materials of this paper and in the PyGMQL GitHub repository. For a progressive introduction to PyGMQL usage, the applications are increasingly complex both for what concerns the biological analysis and the data size Additional file 1.

**Fig. 4:**
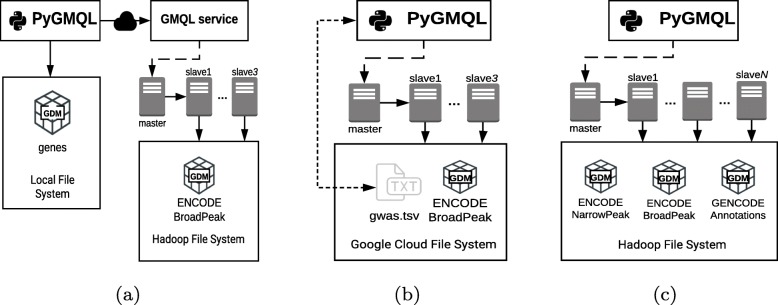
Schematic representation of the deployment strategies adopted in the three applications. **a** Local/Remote system interaction for the analysis of ENCODE histone marks signal on promotorial regions. The gene dataset is stored in the local file system, the ENCODE BroadPeak database is hosted in the GMQL remote repository, deployed on the Hadoop file system with three slaves. **b** Configuration for the interactive analysis of the GWAS dataset against the whole set of enhancers from ENCODE. The library interacts directly with the YARN cluster and the data is stored in the Google Cloud File System with a fixed configuration of three slaves, accessed through the Hadoop engine. The gwas.tsv file is downloaded from the web and stored in the file system before executing the query. **c** Distributed setup for running the TICA query. Three datasets (from ENCODE and GENCODE) are in GDM format and stored in HDFS and the query runs on Amazon Web Services with a variable number of slave nodes, for evaluating the scalability of the system

Examples show: (a) the interplay of local and remote GMQL resources, (b) the scalability with datasets of increasing size. A schematic description of the three deployment strategies is shown in Fig. 4 together with the location of the datasets used during the analysis. We will focus on the system’s scalability in the third and most complex example.

Examples show the interplay between pure Python code and scalable genomic operations, implemented in PyGMQL through the GMQL engine. For this reason, a complete implementation of the examples in GMQL is not possible. While it is possible to implement them by stacking together some of the previously cited tools or even standalone Python code, their parallelization and metadata management would induce a great development overhead for the researcher. The examples highlight the expressiveness, cleanness and ease to use of the library, demonstrating that complex parallel genomic data analysis workflows can be achieved using just PyGMQL.

### Interfacing with an external GMQL service: aggregating the chip-Seq signal of histone Marks on promotorial regions

In this first application, genes’ promoters are extracted from a local dataset and a large set of Chip-Seq experiments is selected from a remote repository. Then, for every promoter and for every Chip-seq experiment, the average signal of those Chip-Seq peaks intersecting the promoter is computed. The result is finally visualized as a heatmap, with rows representing promoters and columns representing Chip-Seq experiments.

This example shows: (i) the integration of local PyGMQL programs with remote repositories, (ii) the possibility to outsource the execution to an external deployment of (Py)GMQL, (iii) the interplay between PyGMQL data and Python libraries written by third parties. These features allow users to write arbitrary complex queries - whose execution and size of the inputs exceed the capabilities of the local environment - and, at the same time, analyze/visualize the output by means of well-known Python libraries.

The code begins by loading a local dataset of gene annotations and extracting their promotorial regions (here defined as regions at [*gene*_*start*_−2000;*gene*_*start*_+2000]). Note that the “.start” and “.stop” attributes automatically consider the strand of the region.


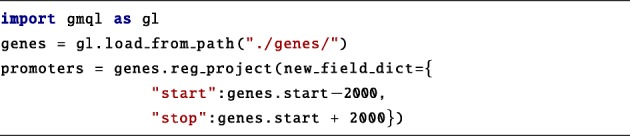


The genes and promoters variables are GMQLDataset, the former is loaded directly, the latter results from a projection operation. Region feature names can be accessed directly from variables to build expressions and predicates (e.g., gene.start + 2000).

Next, we load the external dataset of Chip-Seq from a remote GMQL Web service. In order to do so, the user has to specify the remote address and login. If the user has already signed to the remote GMQL installation, he/she can use his/her own credentials (this will also grant the access to private datasets), otherwise a guest account is automatically created, without requiring the user to do it manually.


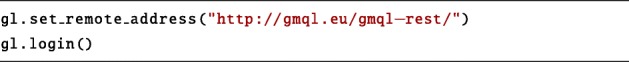


Next, we show how to load the Chip-Seq data of the ENCODE dataset from the remote GMQL repository and select only the experiments of interest. First, the user sets the remote execution mode and imports remote datasets with the load_from_remote function. Such loading is *lazy*, therefore no actual data is moved or red at this point. Then the user specifies the select condition: the hms[~experiment_target~] notation enables the user to build predicates on the given metadata attribute. The GMQL engine loads from the dataset only the samples whose metadata satisfy such condition, specifically, only experiments targeting the *human H3K9ac marker* will be selected.


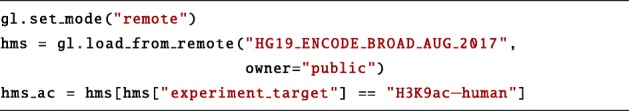


Next, the PyGMQL map operation is used to compute the average of the signal of hms_ac intersecting each promoter: iteration over all samples is implicit. Finally, the materialize method triggers the execution of the query. Since the mode is set to "remote", the dataset stored at ~./genes/~ is sent to the remote service GMQL system that performs the specified operations. The result is loaded into the mappingGDataframe variable which resides on the local machine.


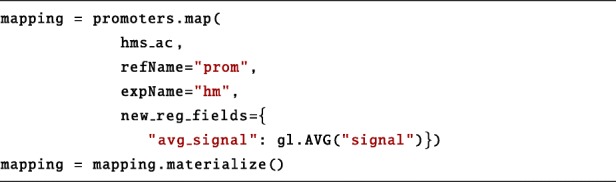


At this point, Python libraries for data manipulation, visualization or analysis can be applied to the GDataframe. The following portion of code provides an example of data manipulation of a query result. The to_matrix method transforms the GDataframe into a Pandas matrix, where each row corresponds to a gene and each column to a cell line. Values are the average signal on the promoter of the given gene in the given cell line. Finally, the matrix is visualized as a heatmap.


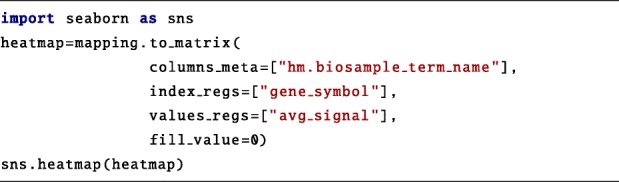


### Exploring data interactively: analyzing GWAS mutations on cell-specific enhancers

In the following example, we explore the interaction between a Genome-wide Association Study (GWAS) dataset (downloaded from an external source^5^) and tracks from the ENCODE dataset, stored in the distributed file system. PyGMQL is deployed on a small Hadoop Cluster. GWAS (genome-wide association studies) are associations between mutations and the traits of individuals carrying the mutations. The following example is inspired by a published research connecting mutations occurring on enhancers (regulatory regions of the genome) with autoimmune diseases [29]. The scientist loads a set of GWAS and investigates if they overlap with enhancer regions which are *cell-specific*, i.e., are active only in a limited number of cell lines or tissues (typically one or two). The interesting aspect of this query is that some of the discovered traits are associated with that cell line, e.g., because they are concerned with a disease affecting the tissue of the cell line.

For a given GWAS dataset, considering all cell lines available in ENCODE, we first extract cell-specific enhancers, then we intersect GWAS with them, and then we count the number of variants which are associated with each trait, and rank traits by such counter. For the first ranked traits, we then represent a heat map having cell lines as rows and traits as columns.

In the Supplementary Materials and on the project web page we present the full data exploration. We next show the main steps and results, assuming that the user has already downloaded the GWAS dataset and stored it in HDFS, where the GMQL repository is also accessible. By default, the library sets the master node of computation to the local Scala back-end. In this case, we deploy the library to a Spark cluster managed by the YARN resource manager [30]. Therefore, we need to use the set_master function, which will request PyGMQL to open a connection to the YARN master node and submit its back-end code for computation.


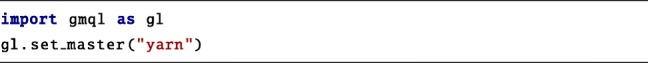


We then load the GWAS dataset, which is encoded as a Tab-separated values file (TSV). Note that, in addition to GDM-like datasets, PyGMQL can load data with generic format using the load_from_file function. In order to do so, the user must configure a custom parser for the TSV format, by specifying the position of the chromosome and the start/end positions (in this particular case the 11-th and 12-th columns respectively) and the position of any of the fields he/she wishes to import (in this example, the 7-th column, which stores the indication of the disease/trait associated with the mutation). The file is then imported as a valid GDM instance. To explore instances, we use the head function, that displays the first rows of both regions and metadata. Its effect is shown in the Supplementary Material. 
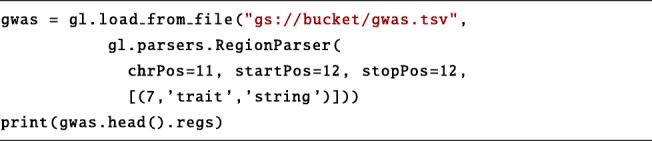


We next load the ENCODE Broad Peak and select from it the Chip-Seq experiments of H3K27ac, as predictor of enhancer activity. This is done with a selection on the experiment_target metadata attribute. We perform a normalization by extracting the position in the middle of each Chip-Seq region with a reg_project operation and then by extending the mid position of a given base pair interval on both sides, again with a projection. We set *interval* to ±1500 bp, resulting in enlarge regions of 3000 base pair length.


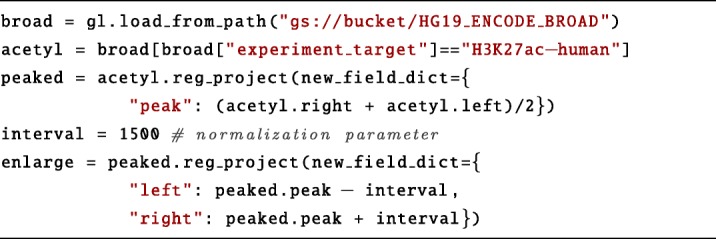


To select only cell-specific enhancers, we need to perform several region manipulation operations.

- First, we group the enlarge regions by the cell line on which they were probed. We also merge overlapping regions of the same cell line which could be due to replicas. This is done with a normal_cover operation adding the groupBy clause on the biosample_term_name metadata attribute, which specifies the cell line of origin for every sample.
- Next, we use again the normal_cover operation to filter cell-specific enhancer regions, i.e. regions that are present in more than a specified maximum number of cell lines (max_overlapping variable). This parameter can be set to small numbers, such as 1 or 2, to increase specificity.
- Finally, we intersect the set of cell-specific enhancers produced by the second cover operation with the set of enhancers grouped by cell line (join with distance less than zero basis, *DLE*(0)). This results in a dataset with a sample for each cell line containing its set of specific enhancers.

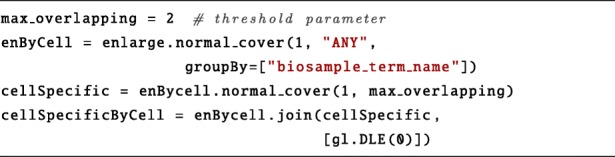


As final operation, we aggregate the GWAS mutations on each cell-specific enhancer region. This is easily done using the map operation projecting the gwas onto cell-specific enhancers. We also want to keep track of the list of diseases/traits associated to each enhancer region, thus we create a new region attribute ’traits’ with the BAG operator on the ’trait’ attribute of gwas.

Query computation on the cluster is triggered by the materialization of the result in the resultGDataframe variable.


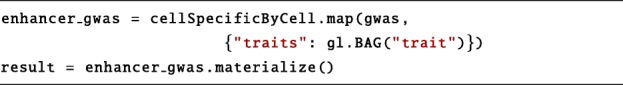


The rest of the analysis can be now done directly in Python, since the result size is typically manageable. We refer the reader to the Supplementary Material for what concerns the operations required to obtain the final heatmap. This analysis can be repeated for arbitrary GWAS databases, changing the setting of interval and max_overlapping, thereby repeating explorations seeking for results of desired cardinality and specificity.

### Performing large queries in the cloud: transcription factors interaction analyzer

An interesting aspect of epigenomic research is concerned with the interaction of transcription factors, i.e. proteins that, once bound onto specific positions of the DNA, enhance or repress the transcription of genes into RNA. Transcription factors (TFs) are known to act in cooperation, as a functional complex. In previous work, we developed TICA (Transcriptional Interaction and Co-regulation Analyser) and an associated Web service [31, 32] using GMQL. TICA is able to predict whether two TFs cooperate to the regulation of the expression of genes by forming a protein complex.

The data-intensive computation has been ported to PyGMQL. The purpose of this Section is not to show the PyGMQL code - as it is rather complex, see Supplementary Material - but rather to show that it scales to very large datasets. In essence, for every cell line the code considers all possible pairs of TFs for which ChIP-seq experiments are available (ranging between 116 in GM12878 and 268 in K562) as candidate complexes. For each such pair, it computes the bindings which are at minimal distance within promotorial regions. Once minimal distance pairs are extracted by PyGMQL, a significance test (written in Python) predicts if the two TFs form a complex.

The code reported in the Supplementary Material illustrates the data extraction part. It uses a normal_cover so as to merge replicates of the same experiment and then two join operations, the former for detecting the overlap between each TF region and active promotorial regions, the latter for extracting the pairs of regions of two TFs at minimal distance within such regions. Joins are complex operations, and here they are performed in the three-dimensional full genome space formed by the bindings of the two TFs and of the promotorial regions.

## Performance evaluation

The ability to scale on parallel and cloud computing environments is the most innovative and distinguishing feature of PyGMQL and allows performing complex queries on large datasets. In this Section, we show the computational performance of PyGMQL on the last application, for three cell lines (GM12878, HepG2, K562) with increasing amount of available samples. For our experiments, we used AWS Elastic MapReduce clusters of *m5.2xlarge* instances, each of which has 8 virtual cores, 32 GiB of memory and 300 GiB of hard disk capacity. We used 4 different setups, all with 1 single master nodes and 1, 3, 5 and 10 slaves.

**Fig. 5:**
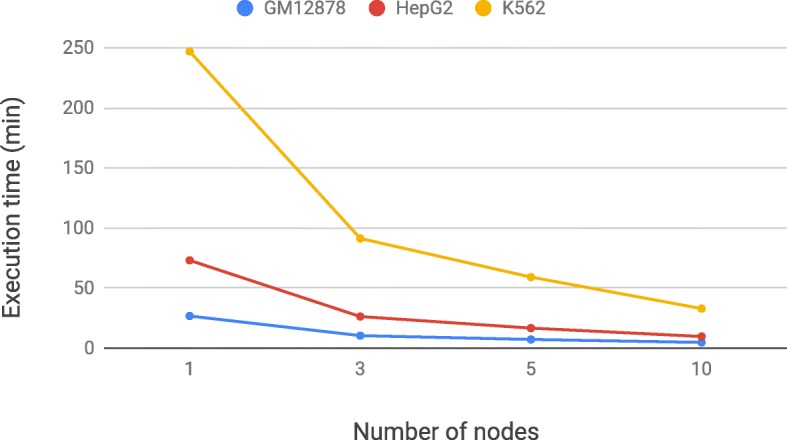
Execution time for the TICA query on three different cell lines, with four different cluster configurations

Table 2 reports the data size of input and output of the three variant of the query as well as the execution times on the 4 cluster configurations. Figure 5 demonstrates the scalability of PyGMQL. Indeed, the execution times decrease as the size of the cluster increases. This effect is especially observable in the K562 and HepG2 cases (the biggest ones), for which the execution is approximately 7.5 times faster in the cluster with 10 slaves with respect to the cluster with one slave. By using the cluster with 10 slaves, we build output data of 381M regions in about half hour, enabling us to compute prediction estimates for the cooperation of about 72K pairs of TFs.

**Table 2:** Sizes of inputs ad outputs for three different cell lines, and execution times (in minutes) for the TICA query over four cluster configurations

	GM12878	HepG2	K562
Input samples	164	224	347
Distinct TFs	116	192	268
Input regions	3,003,121	4,384,181	6,101,933
Output samples	13,454	36,330	71.612
Output regions	109,858,355	213,499,617	381,255,507
Output size (MB)	3,122	6,064.	10,921
1 node e. t. ^∗^	26.73	73.05	246.85
3 nodes e. t. ^∗^	10.40	26.28	91.27
5 nodes e. t. ^∗^	7.21	16.67	59.12
10 nodes e. t. ^∗^	4.75	9.67	32.92

## Conclusions

Python is becoming the leading programming language for data science, thanks to its flexibility and ease of use, the embedding within Jupyter notebooks, and the huge number of supporting libraries and packages. Within the scope of genomic computing, PyGMQL is a new Python library for linking domain-specific data extraction to domain-independent tools and environments for data analysis and visualization.

PyGMQL fills the gap between the scalable Spark-based data management engine of GMQL and the huge body of Python-based resources. PyGMQL supports data interoperability, solves the impedance mismatch between set-oriented data extraction and imperative programming, provides distribution transparency and query outsourcing to powerful server-based and cloud-based systems. The possibility of supporting both local and remote queries enables the efficient prototyping of data extraction pipelines, which can be locally developed and then deployed to big remote services.

## Availability of source code and requirements

- Project name: PyGMQL
- Project home page: https://github.com/DEIB-GECO/PyGMQL
- Operating system(s): Platform independent
- Programming language: Python (version 3.4 or higher)
- Other requirements: Java 1.8 or higher
- License: Apache 2.0
- Any restrictions to use by non-academics: None

## Supplementary information

**Additional file 1** Supplementary materials guide. Document describing how to reproduce the pipelines presented in the manuscript (PDF).

**Additional file 2** Library documentation. Detailed documentation of the software package (PDF).

## Data Availability

Additional file 1 describes how to reproduce the experiments that we present in the manuscript. We provide a Docker image to facilitate the reproducibility of the examples at https://hub.docker.com/r/gecopolimi/pygmql. Source code of the library can be found at https://github.com/DEIB-GECO/PyGMQL.

## Abbreviations

GATK: Genome analysis toolkit
GDM: Genomic data model
GMQL: Genometric query language
GWAS: Genome-wide association study
HDFS: Hadoop file system
TF: Transcription factor
TICA: Transcriptional interaction and co-regulation analyser
TSV: Tab-separated values file
YARN: Yet another resource negotiator

## Footnotes

1 https://github.com/hail-is/hail/

2 Two workspaces describing how to use and develop pipelines built with GMQL and PyGMQL are publicly available to Terra users at https://terra.bio/

3 https://pandas.pydata.org/

4 https://jupyter.org/

5 https://www.ebi.ac.uk/gwas/docs/file-downloads
