# Supplementary material for: PyGMQL: scalable data extraction and analysis for heterogeneous genomic datasets
Source: BMC Bioinformatics. 2019 Nov 8;20:560. doi: 10.1186/s12859-019-3159-9 (PMC6842186; doi:10.1186/s12859-019-3159-9)
Supplement: Supplementary file 1 — Additional file 1 Supplementary materials guide. Document describing how to reproduce the pipelines presented in the manuscript (PDF). [file 12859_2019_3159_MOESM1_ESM.pdf]

PyGMQL Documentation

Release 0.1.2

Luca Nanni
Pietro Pinoli
Arif Canakoglu
Stefano Ceri

Sep 10, 2019

Contents:

1 Installation 3

2 Introduction 5

3 The Genomic data model 9

4 Genometric Query Language 11

5 The GMQLDataset 13

6 Building expressions 25

7 GDataframe 27

8 Remote data management 29

9 Library settings 33

10 Spark and system configurations 35

11 Tutorials 37

12 Data structures and functions 39

Python Module Index 43

Index 45

PyGMQL is a python module that enables the user to perform operation on genomic data in a scalable way.

This library is part of the bigger project GMQL which aims at designing and developing a genomic data management and analysis software on top of big data engines for helping biologists, researchers and data scientists.

GMQL is a declarative language with a SQL-like syntax. PyGMQL translates this paradigm to the interactive and script-oriented world of python, enabling the integration of genomic data with classical Python packages for machine learning and data science.

CHAPTER 1

Installation

1.1 Prerequisites

Here we list the requirements of this library from the point of view of the Python versions that are supported and the external programs needed in order to use it.

1.1.1 Java

In order to use the library you need to have Java installed in your system. And, in particular, the environment variable JAVA_HOME must be setted to your current Java installation.

If JAVA_HOME is not setted an error will be thrown at the first import of the library. In that case the following steps must be perfomed:

1. Install the latest version of Java (follows this link)
2. Set JAVA_HOME. This can be done differently depending on the your OS:
    1. Linux:

        ```
        echo export "JAVA_HOME=/path/to/java" >> ~/.bash_profile
        source ~/.bash_profile
        ```

    2. Mac:

        ```
        echo export "JAVA_HOME=\$(/usr/libexec/java_home)" >> ~/.bash_profile
        source ~/.bash_profile
        ```

    3. Windows:
        1. Right click My Computer and select Properties
        2. On the Advanced tab, select Environment Variables, and then edit JAVA_HOME to point to where the JDK software is located, for example, C:\Program Files\Java\jdk1.6.0_02

1.1.2 Python

Currently PyGMQL supports only Python 3.5, 3.6 and 3.7.

1.2 Using the github repository

You can install this library by downloading its source code from the github repository:

```
git clone https://github.com/DEIB-GECO/PyGMQL.git
```

and then using:

```
cd PyGMQL/
pip install -e .
```

This will install the library and its dependencies in your system.

1.3 Using PIP

The package can be also downloaded and installed directly in your python distribution using:

```
pip install gmql
```

1.4 Installation of the backend

PyGMQL computational engine is written in Scala. The backend comes as a JAR file which will be downloaded at the first usage of the library:

```
import gmql
```

CHAPTER 2

Introduction

In this brief tutorial we will explain the typical workflow of the user of PyGMQL. In the github page of the project you can find a lot more example of usage of the library.

You can use this library both interactively and programmatically. **We strongly suggest to use it inside a Jupyter notebook for the best graphical render and data exploration.**

2.1 Importing the library

To import the library simply type:

```
import gmql as gl
```

If it is the first time you use PyGMQL, the Scala backend program will be downloaded. Therefore we suggest to be connected to the internet the first time you use the library.

2.2 Loading of data

The first thing we want to do with PyGMQL is to load our data. You can do that by calling the *gmql.dataset.loaders.Loader.load_from_path()*. This method loads a reference to a local gmql dataset in memory and creates a *GMQLDataset*. If the dataset in the specified directory is already GMQL standard (has the xml schema file), you only need to do the following:

```
dataset = gl.load_from_path(local_path="path/to/local/dataset")
```

while, if the dataset has no schema, you need to provide it manually. This can be done by creating a custom parser using *RegionParser* like in the following:

```
custom_parser = gl.parsers.RegionParser(chrPos=0, startPos=1, stopPos=2,
                                        otherPos=[(3, "gene", "string")])
dataset = gl.load_from_path(local_path="path/to/local/dataset", parser=custom_parser)
```

2.3 Writing a GMQL query

Now we have a dataset. We can now perform some GMQL operations on it. For example, we want to select samples that satisfy a specific metadata condition:

```
selected_samples = dataset[ (dataset['cell'] == 'es') | (dataset['tumor'] == 'brca') ]
```

Each operation on a GMQLDataset returns an other GMQLDataset. You can also do operations using two datasets:

```
other_dataset = gl.load_from_path("path/to/other/local/dataset")

union_dataset = dataset.union(other_dataset)                        # the union
join_dataset = dataset.join(other_dataset, predicate=[gl.MD(10000)])    # a join
```

2.4 Materializing a result

PyGMQL implements a lazy execution strategy. No operation is performed until a materialize operation is requested:

```
result = join_dataset.materialize()
```

If nothing is passed to the materialize operation, all the data are directly loaded in memory without writing the result dataset to the disk. If you want also to save the data for future computation you need to specify the output path:

```
result = join_dataset.materialize("path/to/output/dataset")
```

2.5 The result data structure

When you materialize a result, a `GDataframe` object is returned. This object is a wrapper of two pandas dataframes, one for the regions and one for the metadata. You can access them in the following way:

```
result.meta     # for the metadata
result.regs     # for the regions
```

These dataframes are structured as follows:

- The region dataframe puts in every line a different region. The source sample for the specific region is the index of the line. Each column represent a field of the region data.
- The metadata dataframe has one row for each sample in the dataset. Each column represent a different metadata attribute. Each cell of this dataframe represent the values of a specific metadata for that specific sample. Multiple values are allowed for each cell.

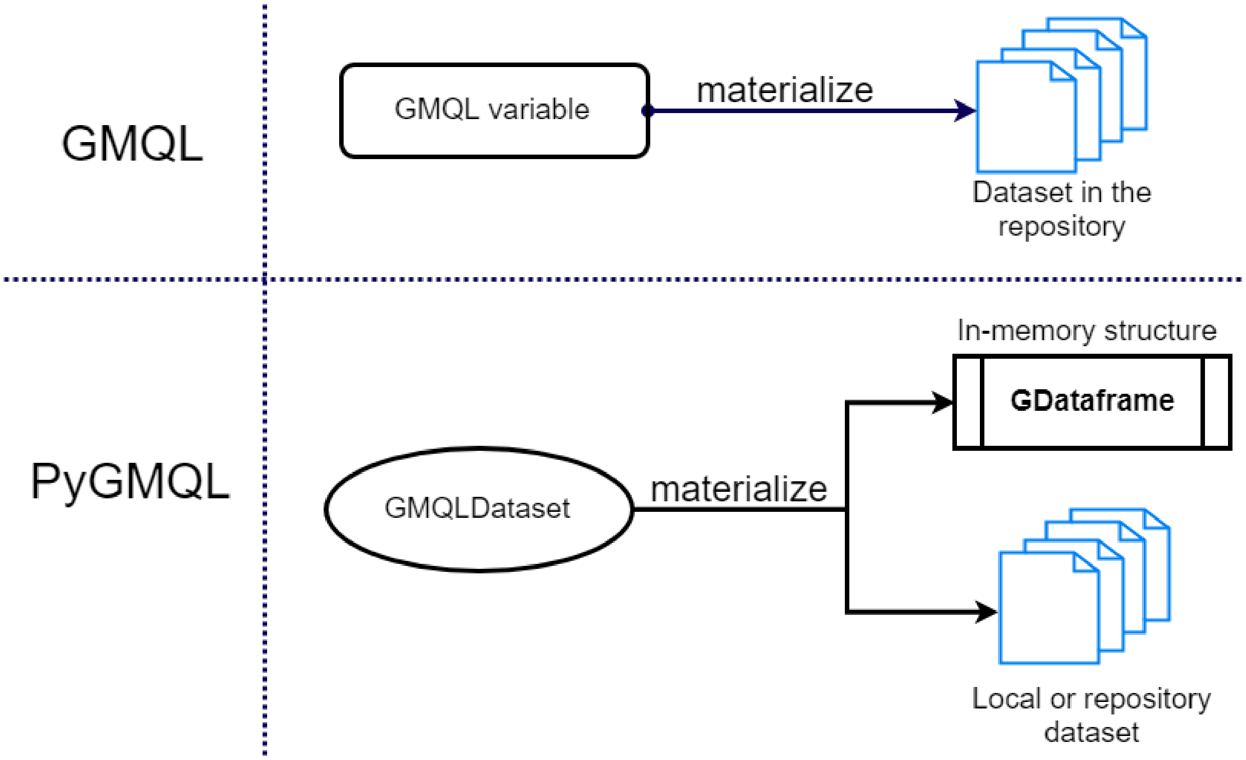
GMQL
GMQL variable
materialize
Dataset in the repository
PyGMQL
In-memory structure
GDataframe
GMQLDataset
materialize
Local or repository dataset

CHAPTER 3

The Genomic data model

As we have said, PyGMQL is a Python interface to the GMQL system. In order to understand how the library works, a little insights on the data model used by GMQL is necessary.

GMQL is based on a representation of the genomic information known as GDM - Genomic Data Model. Datasets are composed of samples , which in turn contains two kinds of data:

- **Region values** (or simply **regions**), aligned w.r.t. a given reference, with specific left-right ends within a chromosome. Regions can store different information regarding the "spot" they mark in a particular sample, such as region length or statistical significance. Regions of the model describe processed data, e.g. mutations, expression or bindings; they have a **schema** , with 5 common attributes (id , chr , left , right , strand) including the id of the region and the region coordinates, along the aligned reference genome, and then arbitrary typed attributes. This provides interoperability across a plethora of genomic data formats
- **Metadata**, storing all the knowledge about the particular sample, are arbitrary attribute-value pairs, independent from any standardization attempt; they trace the data provenance, including biological and clinical aspects

This is exemplified by the figure below, showing on the left the regions and on the right the metadata of a dataset sample.

ID,	Chr,	Start,	Stop,	Strand,	[p-value]
1	chr1	10	20	*	0.00025
1	chr1	30	40	*	0.00057
1	chr1	45	65	*	0.015
2	chr1	15	25	*	0.01204
2	chr1	30	50	*	0.0002

ID,	Attribute,	Value
1	antibody_target	H3K4me1
1	cell	K562
1	data_type	Chip-seq
2	antibody_target	CTCF
2	cell	K562

CHAPTER 4

Genometric Query Language

GMQL is a declarative language for genomic region and metadata manipulation with a SQL-inspired syntax. With GMQL the user can perform complex queries on the basis of positional, categorical and numeric features of the datasets.

You can find more information about the language at the following links:

- Complete introduction to the GMQL language: here we explain all the operators of the GMQL language together with some explanatory examples.
- Explained examples and biological applications: here we present several complex queries showing the expressiveness of the language in a biological research setting.

NB: In order to use PyGMQL one should have at least clear the semantics of the GMQL operators, but the library is designed to be self contained and can be used without a strong background knowledge of the language.

4.1 GMQL engine

The GMQL engine is composed by various sub-systems:

- *A repository*, which enables the user to store his/her datasets, the results of the queries and to access the public datasets shared between the users of the same GMQL instance
- *An engine implementation*, which implements the GMQL operators. Currently the Spark engine is the most updated and complete implementation and it is the one used also by PyGMQL

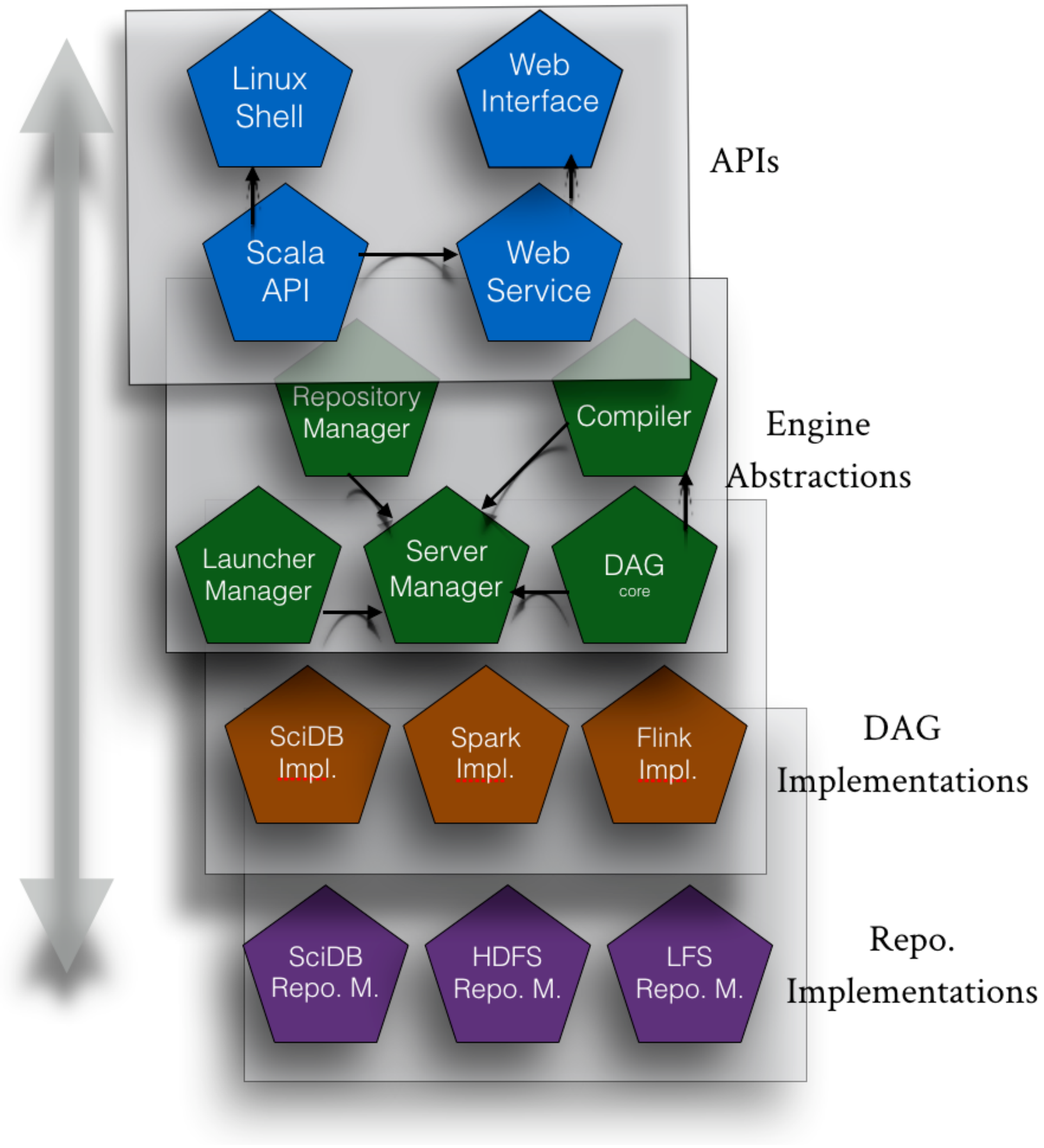

4.2 GMQL WEB interface

The GMQL system is publicly available at this link.

CHAPTER 5

The GMQLDataset

Here we present the functions that can be used on a GMQLDataset.

class GMQLDataset(*parser=None, index=None, location='local', path_or_name=None, local_sources=None, remote_sources=None, meta_profile=None*)

The main abstraction of the library. A GMQLDataset represents a genomic dataset in the GMQL standard and it is divided in region data and meta data. The function that can be applied to a GMQLDataset affect one of these two features or both.

For each operator function that can be applied to a GMQLDataset we provide the documentation, some examples, and we specify which operator of GMQL the function is wrapper of.

get_reg_attributes()

Returns the region fields of the dataset

Returns a list of field names

MetaField(*name, t=None*)

Creates an instance of a metadata field of the dataset. It can be used in building expressions or conditions for projection or selection. Notice that this function is equivalent to call:

```
dataset["name"]
```

If the MetaField is used in a region projection (*reg_project()*), the user has also to specify the type of the metadata attribute that is selected:

```
dataset.reg_project(new_field_dict={'new_field': dataset['name', 'string']})
```

Parameters

- **name** – the name of the metadata that is considered
- **t** – the type of the metadata attribute {string, int, boolean, double}

Returns a MetaField instance

RegField(*name*)
Creates an instance of a region field of the dataset. It can be used in building expressions or conditions for projection or selection. Notice that this function is equivalent to:

```
dataset.name
```

Parameters **name** – the name of the region field that is considered

Returns a RegField instance

select(*meta_predicate=None*, *region_predicate=None*, *semiJoinDataset=None*, *semiJoinMeta=None*)
Wrapper of `SELECT`

Selection operation. Enables to filter datasets on the basis of region features or metadata attributes. In addition it is possibile to perform a selection based on the existence of certain metadata `semiJoinMeta` attributes and the matching of their values with those associated with at least one sample in an external dataset `semiJoinDataset`.

Therefore, the selection can be based on:

- *Metadata predicates*: selection based on the existence and values of certain metadata attributes in each sample. In predicates, attribute-value conditions can be composed using logical predicates & (and), | (or) and ~ (not)
- *Region predicates*: selection based on region attributes. Conditions can be composed using logical predicates & (and), | (or) and ~ (not)
- *SemiJoin clauses*: selection based on the existence of certain metadata `semiJoinMeta` attributes and the matching of their values with those associated with at least one sample in an external dataset `semiJoinDataset`

In the following example we select all the samples from Example_Dataset_1 regarding antibody CTCF. From these samples we select only the regions on chromosome 6. Finally we select only the samples which have a matching antibody_targetClass in Example_Dataset_2:

```
import gmql as gl
d1 = gl.get_example_dataset("Example_Dataset_1")
d2 = gl.get_example_dataset("Example_Dataset_2")

d_select = d.select(meta_predicate = d['antibody'] == "CTCF",
                    region_predicate = d.chr == "chr6",
                    semiJoinDataset=d2, semiJoinMeta=["antibody_targetClass"])
```

Parameters

- **meta_predicate** – logical predicate on the metadata <attribute, value> pairs
- **region_predicate** – logical predicate on the region feature values
- **semiJoinDataset** – an other GMQLDataset
- **semiJoinMeta** – a list of metadata attributes (strings)

Returns a new GMQLDataset

meta_select(*predicate=None*, *semiJoinDataset=None*, *semiJoinMeta=None*)
Wrapper of `SELECT`

Wrapper of the *`select()`* function filtering samples only based on metadata.

Parameters

- **predicate** – logical predicate on the values of the rows
- **semiJoinDataset** – an other GMQLDataset
- **semiJoinMeta** – a list of metadata

Returns a new GMQLDataset

Example 1:

```
output_dataset = dataset.meta_select(dataset['patient_age'] < 70)
# This statement can be written also as
output_dataset = dataset[ dataset['patient_age'] < 70 ]
```

Example 2:

```
output_dataset = dataset.meta_select( (dataset['tissue_status'] == 'tumoral')␣
↪&
                                      (tumor_tag != 'gbm') | (tumor_tag == 'brca
↪'))
# This statement can be written also as
output_dataset = dataset[ (dataset['tissue_status'] == 'tumoral') &
                          (tumor_tag != 'gbm') | (tumor_tag == 'brca') ]
```

Example 3:

```
JUN_POLR2A_TF = HG19_ENCODE_NARROW.meta_select( JUN_POLR2A_TF['antibody_target
↪'] == 'JUN',
                                                semiJoinDataset=POLR2A_TF,␣
↪semiJoinMeta=['cell'])
```

The meta selection predicate can use all the classical equalities and disequalities {>, <, >=, <=, ==, !=} and predicates can be connected by the classical logical symbols {& (AND), | (OR), ~ (NOT)} plus the *isin* function.

reg_select (*predicate=None*, *semiJoinDataset=None*, *semiJoinMeta=None*)

Wrapper of `SELECT`

Wrapper of the `select()` function filtering regions only based on region attributes.

Parameters

- **predicate** – logical predicate on the values of the regions
- **semiJoinDataset** – an other GMQLDataset
- **semiJoinMeta** – a list of metadata

Returns a new GMQLDataset

An example of usage:

```
new_dataset = dataset.reg_select((dataset.chr == 'chr1') | (dataset.pValue <␣
↪0.9))
```

You can also use Metadata attributes in selection:

```
new_dataset = dataset.reg_select(dataset.score > dataset['size'])
```

This statement selects all the regions whose field score is strictly higher than the sample metadata attribute size.

The region selection predicate can use all the classical equalities and disequalities {>, <, >=, <=, ==, !=} and predicates can be connected by the classical logical symbols {& (AND), | (OR), ~ (NOT)} plus the *isin* function.

In order to be sure about the correctness of the expression, please use parenthesis to delimit the various predicates.

project (*projected_meta=None*, *new_attr_dict=None*, *all_but_meta=None*, *projected_regs=None*, *new_field_dict=None*, *all_but_regs=None*)
Wrapper of `PROJECT`

The PROJECT operator creates, from an existing dataset, a new dataset with all the samples (with their regions and region values) in the input one, but keeping for each sample in the input dataset only those metadata and/or region attributes expressed in the operator parameter list. Region coordinates and values of the remaining metadata and region attributes remain equal to those in the input dataset. Differently from the SELECT operator, PROJECT allows to:

- Remove existing metadata and/or region attributes from a dataset;
- Create new metadata and/or region attributes to be added to the result.

Parameters

- **projected_meta** – list of metadata attributes to project on
- **new_attr_dict** – an optional dictionary of the form {'new_meta_1': function1, 'new_meta_2': function2, . . . } in which every function computes the new metadata attribute based on the values of the others
- **all_but_meta** – list of metadata attributes that must be excluded from the projection
- **projected_regs** – list of the region fields to select
- **new_field_dict** – an optional dictionary of the form {'new_field_1': function1, 'new_field_2': function2, . . . } in which every function computes the new region field based on the values of the others
- **all_but_regs** – list of region fields that must be excluded from the projection

Returns a new GMQLDataset

meta_project (*attr_list=None*, *all_but=None*, *new_attr_dict=None*)
Wrapper of `PROJECT`

Project the metadata based on a list of attribute names

Parameters

- **attr_list** – list of the metadata fields to select
- **all_but** – list of metadata that must be excluded from the projection.
- **new_attr_dict** – an optional dictionary of the form {'new_field_1': function1, 'new_field_2': function2, . . . } in which every function computes the new field based on the values of the others

Returns a new GMQLDataset

Notice that if attr_list is specified, all_but cannot be specified and viceversa.

Examples:

```
new_dataset = dataset.meta_project(attr_list=['antibody', 'ID'],
                                   new_attr_dict={'new_meta': dataset['ID'] +␣
↪100})
```

(continues on next page)

(continued from previous page)

reg_project (*field_list=None*, *all_but=None*, *new_field_dict=None*)
Wrapper of PROJECT

Project the region data based on a list of field names

Parameters

- **field_list** – list of the fields to select
- **all_but** – keep only the region fields different from the ones specified
- **new_field_dict** – an optional dictionary of the form {'new_field_1': function1, 'new_field_2': function2, . . . } in which every function computes the new field based on the values of the others

Returns a new GMQLDataset

An example of usage:

```
new_dataset = dataset.reg_project(['pValue', 'name'],
                                  {'new_field': dataset.pValue / 2})

new_dataset = dataset.reg_project(field_list=['peak', 'pvalue'],
                                  new_field_dict={'new_field': dataset.pvalue␣
↪* dataset['cell_age', 'float']})
```

Notice that you can use metadata attributes for building new region fields. The only thing to remember when doing so is to define also the type of the output region field in the metadata attribute definition (for example, `dataset['cell_age', 'float']` is required for defining the new attribute `new_field` as float). In particular, the following type names are accepted: 'string', 'char', 'long', 'integer', 'boolean', 'float', 'double'.

extend (*new_attr_dict*)
Wrapper of EXTEND

For each sample in an input dataset, the EXTEND operator builds new metadata attributes, assigns their values as the result of arithmetic and/or aggregate functions calculated on sample region attributes, and adds them to the existing metadata attribute-value pairs of the sample. Sample number and their genomic regions, with their attributes and values, remain unchanged in the output dataset.

Parameters **new_attr_dict** – a dictionary of the type {'new_metadata' : AGGREGATE_FUNCTION('field'), . . . }

Returns new GMQLDataset

An example of usage, in which we count the number of regions in each sample and the minimum value of the *pValue* field and export it respectively as metadata attributes *regionCount* and *minPValue*:

```
import gmql as gl

dataset = gl.get_example_dataset("Example_Dataset_1")
new_dataset = dataset.extend({'regionCount' : gl.COUNT(),
                              'minPValue' : gl.MIN('pValue')})
```

cover (*minAcc*, *maxAcc*, *groupBy=None*, *new_reg_fields=None*, *cover_type='normal'*)
Wrapper of COVER

COVER is a GMQL operator that takes as input a dataset (of usually, but not necessarily, multiple samples) and returns another dataset (with a single sample, if no groupby option is specified) by "collapsing"

the input samples and their regions according to certain rules specified by the COVER parameters. The attributes of the output regions are only the region coordinates, plus in case, when aggregate functions are specified, new attributes with aggregate values over attribute values of the contributing input regions; output metadata are the union of the input ones, plus the metadata attributes JaccardIntersect and JaccardResult, representing global Jaccard Indexes for the considered dataset, computed as the correspondent region Jaccard Indexes but on the whole sample regions.

Parameters

- **cover_type** – the kind of cover variant you want ['normal', 'flat', 'summit', 'histogram']
- **minAcc** – minimum accumulation value, i.e. the minimum number of overlapping regions to be considered during COVER execution. It can be any positive number or the strings {'ALL', 'ANY'}.
- **maxAcc** – maximum accumulation value, i.e. the maximum number of overlapping regions to be considered during COVER execution. It can be any positive number or the strings {'ALL', 'ANY'}.
- **groupBy** – optional list of metadata attributes
- **new_reg_fields** – dictionary of the type {'new_region_attribute' : AGGREGATE_FUNCTION('field'), ... }

Returns a new GMQLDataset

An example of usage:

```
cell_tf = narrow_peak.cover("normal", minAcc=1, maxAcc="Any",
                                groupBy=['cell', 'antibody_target'])
```

normal_cover(*minAcc*, *maxAcc*, *groupBy=None*, *new_reg_fields=None*)

Wrapper of `COVER`

The normal cover operation as described in *cover()*. Equivalent to calling:

```
dataset.cover("normal", ...)
```

flat_cover(*minAcc*, *maxAcc*, *groupBy=None*, *new_reg_fields=None*)

Wrapper of `COVER`

Variant of the function *cover()* that returns the union of all the regions which contribute to the COVER. More precisely, it returns the contiguous regions that start from the first end and stop at the last end of the regions which would contribute to each region of a COVER.

Equivalent to calling:

```
cover("flat", ...)
```

summit_cover(*minAcc*, *maxAcc*, *groupBy=None*, *new_reg_fields=None*)

Wrapper of `COVER`

Variant of the function *cover()* that returns only those portions of the COVER result where the maximum number of regions overlap (this is done by returning only regions that start from a position after which the number of overlaps does not increase, and stop at a position where either the number of overlapping regions decreases or violates the maximum accumulation index).

Equivalent to calling:

```
cover("summit", ...)
```

histogram_cover(*minAcc*, *maxAcc*, *groupBy=None*, *new_reg_fields=None*)
Wrapper of COVER

Variant of the function `cover()` that returns all regions contributing to the COVER divided in different (contiguous) parts according to their accumulation index value (one part for each different accumulation value), which is assigned to the AccIndex region attribute.

Equivalent to calling:

```
cover("histogram", ...)
```

join(*experiment*, *genometric_predicate*, *output='LEFT'*, *joinBy=None*, *refName='REF'*, *expName='EXP'*, *left_on=None*, *right_on=None*)
Wrapper of JOIN

The JOIN operator takes in input two datasets, respectively known as anchor (the first/left one) and experiment (the second/right one) and returns a dataset of samples consisting of regions extracted from the operands according to the specified condition (known as genometric predicate). The number of generated output samples is the Cartesian product of the number of samples in the anchor and in the experiment dataset (if no joinby close if specified). The attributes (and their values) of the regions in the output dataset are the union of the region attributes (with their values) in the input datasets; homonymous attributes are disambiguated by prefixing their name with their dataset name. The output metadata are the union of the input metadata, with their attribute names prefixed with their input dataset name.

Parameters

- **experiment** – an other GMQLDataset
- **genometric_predicate** – a list of Genometric atomic conditions. For an explanation of each of them go to the respective page.
- **output** – one of four different values that declare which region is given in output for each input pair of anchor and experiment regions satisfying the genometric predicate:
  - 'LEFT': outputs the anchor regions from the anchor dataset that satisfy the genometric predicate
  - 'RIGHT': outputs the anchor regions from the experiment dataset that satisfy the genometric predicate
  - 'INT': outputs the overlapping part (intersection) of the anchor and experiment regions that satisfy the genometric predicate; if the intersection is empty, no output is produced
  - 'CONTIG': outputs the concatenation between the anchor and experiment regions that satisfy the genometric predicate, i.e. the output region is defined as having left (right) coordinates equal to the minimum (maximum) of the corresponding coordinate values in the anchor and experiment regions satisfying the genometric predicate
- **joinBy** – list of metadata attributes
- **refName** – name that you want to assign to the reference dataset
- **expName** – name that you want to assign to the experiment dataset
- **left_on** – list of region fields of the reference on which the join must be performed
- **right_on** – list of region fields of the experiment on which the join must be performed

Returns a new GMQLDataset

An example of usage, in which we perform the join operation between Example_Dataset_1 and Example_Dataset_2 specifying than we want to join the regions of the former with the first regions at a minimim

distance of 120Kb of the latter and finally we want to output the regions of Example_Dataset_2 matching the criteria:

```
import gmql as gl

d1 = gl.get_example_dataset("Example_Dataset_1")
d2 = gl.get_example_dataset("Example_Dataset_2")

result_dataset = d1.join(experiment=d2,
                         genometric_predicate=[gl.MD(1), gl.DGE(120000)],
                         output="right")
```

map (*experiment*, *new_reg_fields=None*, *joinBy=None*, *refName='REF'*, *expName='EXP'*)
Wrapper of `MAP`

MAP is a non-symmetric operator over two datasets, respectively called reference and experiment. The operation computes, for each sample in the experiment dataset, aggregates over the values of the experiment regions that intersect with a region in a reference sample, for each region of each sample in the reference dataset; we say that experiment regions are mapped to the reference regions. The number of generated output samples is the Cartesian product of the samples in the two input datasets; each output sample has the same regions as the related input reference sample, with their attributes and values, plus the attributes computed as aggregates over experiment region values. Output sample metadata are the union of the related input sample metadata, whose attribute names are prefixed with their input dataset name. For each reference sample, the MAP operation produces a matrix like structure, called genomic space, where each experiment sample is associated with a row, each reference region with a column, and each matrix row is a vector of numbers - the aggregates computed during MAP execution. When the features of the reference regions are unknown, the MAP helps in extracting the most interesting regions out of many candidates.

Parameters

- **experiment** – a GMQLDataset
- **new_reg_fields** – an optional dictionary of the form {'new_field_1': AGGREGATE_FUNCTION(field), . . . }
- **joinBy** – optional list of metadata
- **refName** – name that you want to assign to the reference dataset
- **expName** – name that you want to assign to the experiment dataset

Returns a new GMQLDataset

In the following example, we map the regions of Example_Dataset_2 on the ones of Example_Dataset_1 and for each region of Example_Dataset_1 we ouput the average Pvalue and number of mapped regions of Example_Dataset_2. In addition we specify that the output region fields and metadata attributes will have the *D1* and *D2* suffixes respectively for attributes and fields belonging the Example_Dataset_1 and Example_Dataset_2:

```
import gmql as gl

d1 = gl.get_example_dataset("Example_Dataset_1")
d2 = gl.get_example_dataset("Example_Dataset_2")

result = d1.map(experiment=d2, refName="D1", expName="D2",
                new_reg_fields={"avg_pValue": gl.AVG("pvalue")})
```

order (*meta=None*, *meta_ascending=None*, *meta_top=None*, *meta_k=None*, *regs=None*, *regs_ascending=None*, *region_top=None*, *region_k=None*)
Wrapper of `ORDER`

The ORDER operator is used to order either samples, sample regions, or both, in a dataset according to a set of metadata and/or region attributes, and/or region coordinates. The number of samples and their regions in the output dataset is as in the input dataset, as well as their metadata and region attributes and values, but a new ordering metadata and/or region attribute is added with the sample or region ordering value, respectively.

Parameters

- **meta** – list of metadata attributes
- **meta_ascending** – list of boolean values (True = ascending, False = descending)
- **meta_top** – "top", "topq" or "topp" or None
- **meta_k** – a number specifying how many results to be retained
- **regs** – list of region attributes
- **regs_ascending** – list of boolean values (True = ascending, False = descending)
- **region_top** – "top", "topq" or "topp" or None
- **region_k** – a number specifying how many results to be retained

Returns a new GMQLDataset

Example of usage. We order Example_Dataset_1 metadata by ascending *antibody* and descending *antibody_class* keeping only the first sample. We also order the resulting regions based on the *score* field in descending order, keeping only the first one also in this case:

```
import gmql as gl

d1 = gl.get_example_dataset("Example_Dataset_1")

result = d1.order(meta=["antibody", "antibody_targetClass"],
                  meta_ascending=[True, False], meta_top="top", meta_k=1,
                  regs=['score'], regs_ascending=[False],
                  region_top="top", region_k=1)
```

difference(*other*, *joinBy=None*, *exact=False*)

Wrapper of DIFFERENCE

DIFFERENCE is a binary, non-symmetric operator that produces one sample in the result for each sample of the first operand, by keeping the same metadata of the first operand sample and only those regions (with their schema and values) of the first operand sample which do not intersect with any region in the second operand sample (also known as negative regions)

Parameters

- **other** – GMQLDataset
- **joinBy** – (optional) list of metadata attributes. It is used to extract subsets of samples on which to apply the operator: only those samples in the current and other dataset that have the same value for each specified attribute are considered when performing the operation
- **exact** – boolean. If true, the the regions are considered as intersecting only if their coordinates are exactly the same

Returns a new GMQLDataset

Example of usage. We compute the exact difference between Example_Dataset_1 and Example_Dataset_2, considering only the samples with same *antibody*:

```
import gmql as gl

d1 = gl.get_example_dataset("Example_Dataset_1")
d2 = gl.get_example_dataset("Example_Dataset_2")

result = d1.difference(other=d2, exact=True, joinBy=['antibody'])
```

union (*other*, *left_name='LEFT'*, *right_name='RIGHT'*)
Wrapper of `UNION`

The UNION operation is used to integrate homogeneous or heterogeneous samples of two datasets within a single dataset; for each sample of either one of the input datasets, a sample is created in the result as follows:

- its metadata are the same as in the original sample;
- its schema is the schema of the first (left) input dataset; new identifiers are assigned to each output sample;
- its regions are the same (in coordinates and attribute values) as in the original sample. Region attributes which are missing in an input dataset sample (w.r.t. the merged schema) are set to null.

Parameters

- **other** – a GMQLDataset
- **left_name** – name that you want to assign to the left dataset
- **right_name** – name tha t you want to assign to the right dataset

Returns a new GMQLDataset

Example of usage:

```
import gmql as gl

d1 = gl.get_example_dataset("Example_Dataset_1")
d2 = gl.get_example_dataset("Example_Dataset_2")

result = d1.union(other=d2, left_name="D1", right_name="D2")
```

merge (*groupBy=None*)
Wrapper of `MERGE`

The MERGE operator builds a new dataset consisting of a single sample having

- as regions all the regions of all the input samples, with the same attributes and values
- as metadata the union of all the metadata attribute-values of the input samples.

A groupby clause can be specified on metadata: the samples are then partitioned in groups, each with a distinct value of the grouping metadata attributes, and the MERGE operation is applied to each group separately, yielding to one sample in the result dataset for each group. Samples without the grouping metadata attributes are disregarded

Parameters **groupBy** – list of metadata attributes

Returns a new GMQLDataset

Example of usage:

```python
import gmql as gl

d1 = gl.get_example_dataset("Example_Dataset_1")
result = d1.merge(['antibody'])
```

group (*meta=None*, *meta_aggregates=None*, *regs=None*, *regs_aggregates=None*, *meta_group_name='_group'*)

Wrapper of `GROUP`

The GROUP operator is used for grouping both regions and/or metadata of input dataset samples according to distinct values of certain attributes (known as grouping attributes); new grouping attributes are added to samples in the output dataset, storing the results of aggregate function evaluations over metadata and/or regions in each group of samples. Samples having missing values for any of the grouping attributes are discarded.

Parameters

- **meta** – (optional) a list of metadata attributes
- **meta_aggregates** – (optional) {'new_attr': fun}
- **regs** – (optional) a list of region fields
- **regs_aggregates** – {'new_attr': fun}
- **meta_group_name** – (optional) the name to give to the group attribute in the metadata

Returns a new GMQLDataset

Example of usage. We group samples by *antibody* and we aggregate the region pvalues taking the maximum value calling the new region field *maxPvalue*:

```python
import gmql as gl

d1 = gl.get_example_dataset("Example_Dataset_1")
result = d1.group(meta=['antibody'], regs_aggregates={'maxPvalue': gl.MAX(
↪"pvalue")})
```

meta_group (*meta*, *meta_aggregates=None*)

Wrapper of `GROUP`

Group operation only for metadata. For further information check `group()`

regs_group (*regs*, *regs_aggregates=None*)

Wrapper of `GROUP`

Group operation only for region data. For further information check `group()`

materialize (*output_path=None*, *output_name=None*, *all_load=True*, *mode=None*)

Wrapper of `MATERIALIZE`

Starts the execution of the operations for the GMQLDataset. PyGMQL implements lazy execution and no operation is performed until the materialization of the results is requestd. This operation can happen both locally or remotely.

- Local mode: if the GMQLDataset is local (based on local data) the user can specify the

Parameters

- **output_path** – (Optional) If specified, the user can say where to locally save the results of the computations.

- **output_name** – (Optional) Can be used only if the dataset is remote. It represents the name that the user wants to give to the resulting dataset on the server
- **all_load** – (Optional) It specifies if the result dataset should be directly converted to a GDataframe (True) or to a GMQLDataset (False) for future local queries.

Returns A GDataframe or a GMQLDataset

head(*n=5*)
Returns a small set of regions and metadata from a query. It is supposed to be used for debugging purposes or for data exploration.

Parameters **n** – how many samples to retrieve

Returns a GDataframe

5.1 Loading functions

You can create a GMQLDataset by loading the data using the following functions:

load_from_file(*path*, *parser: gmql.dataset.parsers.RegionParser.RegionParser*)
Loads a GDM dataset from a single BED-like file.

Parameters

- **path** – location of the file
- **parser** – RegionParser object specifying the parser of the file

Returns a GMQLDataset

load_from_path(*local_path*, *parser=None*)
Loads the data from a local path into a GMQLDataset. The loading of the files is "lazy", which means that the files are loaded only when the user does a materialization (see `materialize()`). The user can force the materialization of the data (maybe for an initial data exploration on only the metadata) by setting the `reg_load` (load in memory the region data), `meta_load` (load in memory the metadata) or `all_load` (load both region and meta data in memory). If the user specifies this final parameter as True, a `GDataframe` is returned, otherwise a `GMQLDataset` is returned

Parameters

- **local_path** – local path of the dataset
- **parser** – the parser to be used for reading the data
- **all_load** – if set to True, both region and meta data are loaded in memory and an instance of GDataframe is returned

Returns A new GMQLDataset or a GDataframe

load_from_remote(*remote_name*, *owner=None*)
Loads the data from a remote repository.

Parameters

- **remote_name** – The name of the dataset in the remote repository
- **owner** – (optional) The owner of the dataset. If nothing is provided, the current user is used. For public datasets use 'public'.

Returns A new GMQLDataset or a GDataframe

CHAPTER 6

Building expressions

When doing a selection (using *meta_select()*, *reg_select()*) or a projection (using *meta_project()*, *reg_project()*) you are required to specify an expression on the metadata or region fields.

An expression can therefore use metadata attributes or region fields. Given a GMQLDataset `dataset`, one can access its **region fields** by typing:

```
dataset.field1
dataset.field2
dataset.chr
dataset.start
...
```

and one can access its **metadata attributes** by typing:

```
dataset['metadata_attribute_1']
dataset['metadata_attribute_2']
dataset['metadata_attribute_3']
...
```

The expressions in PyGMQL can be of two types:

- *Predicate*: a logical condition that enables to select a portion of the dataset. This expression is used in selection. Some example of predicates follow:

  ```
  # region predicate
  (dataset.chr == 'chr1' || dataset.pValue < 0.9)
  # region predicate with access to metadata attributes
  dataset.score > dataset['size']
  ```

  It is possible, based on the function that requires a predicate, to mix region fields and metadata attributes in a region condition. Of course it is not possible to mix metadata and region conditions in a metadata selection (this is due to the fact that to each metadata attribute can be associated multiple values for each region field).

- *Extension*: a mathematical expression describing how to build new metadata or region fields based on the existent ones. Some examples of expression follow:

```
# region expression
dataset.start + dataset.stop
dataset.p_value / dataset.q_value
# metadata expression
dataset['size'] * 8.9
dataset['score'] / dataset['size']
```

It is possible to mix region fields and metadata attributes in region extensions:

```
# region expression using metadata attributes
(dataset.pvalue / 2) + dataset['metadata'] + 1
```

CHAPTER 7

GDataframe

class GDataframe(*regs=None*, *meta=None*)

Class holding the result of a materialization of a GMQLDataset. It is composed by two data structures:

- A table with the *region* data
- A table with the *metadata* corresponding to the regions

to_dataset_files(*local_path=None*, *remote_path=None*)

Save the GDataframe to a local or remote location

Parameters

- **local_path** – a local path to the folder in which the data must be saved
- **remote_path** – a remote dataset name that wants to be used for these data

Returns None

to_GMQLDataset(*local_path=None*, *remote_path=None*)

Converts the GDataframe in a GMQLDataset for later local or remote computation

Returns a GMQLDataset

project_meta(*attributes*)

Projects the specified metadata attributes to new region fields

Parameters **attributes** – a list of metadata attributes

Returns a new GDataframe with additional region fields

to_matrix(*index_regs=None*, *index_meta=None*, *columns_regs=None*, *columns_meta=None*, *values_regs=None*, *values_meta=None*, ***kwargs*)

Transforms the GDataframe to a pivot matrix having as index and columns the ones specified. This function is a wrapper around the pivot_table function of Pandas.

Parameters

- **index_regs** – list of region fields to use as index
- **index_meta** – list of metadata attributes to use as index

- **columns_regs** – list of region fields to use as columns
- **columns_meta** – list of metadata attributes to use as columns
- **values_regs** – list of region fields to use as values
- **values_meta** – list of metadata attributes to use as values
- **kwargs** – other parameters to pass to the pivot_table function

Returns a Pandas dataframe having as index the union of index_regs and index_meta, as columns the union of columns_regs and columns_meta and as values ths union of values_regs and values_meta

CHAPTER 8

Remote data management

PyGMQL can be used in two different ways. The first one (and the most intuitive and classical one) is to use it like any other computational library.

PyGMQL also manages the execution through a remote server (or cluster). In order to use this feature the user needs to login to the remote service before.

The web service offered by the GeCo group at Politecnico di Milano can be found at http://gmql.eu/gmql-rest/

8.1 Loggin in

This can be done by firstly specifying the remote server address:

```
import gmql as gl
gl.set_remote_address("http://gmql.eu/gmql-rest/")
```

and then by logging in into the system:

```
gl.login()
```

From this point on the user will be logged into the remote system.

8.2 Guest users VS Authenticated users

GMQL and PyGMQL enable two different ways to interact with the remote service. The users can be logged as:

- *Guest user*: the user doesn't need to register to the service and a only a limited storage and computational power is available for the queries. The access token (which is automatically handled by the library) will expire after a certain period of inactivity.
- *Authenticated user*: the user needs to register on the web interface before providing username, password and other information. The access token is stored and can be used for an unlimited amount of time.

By default, the sequence of operations that are shown above will log the user as *guest*.

In both cases a folder in the home directory of the user will be created with name `.pygmql` and inside of it there will be a `sessions.xml` file which will store all the active sessions for the user.

8.3 Logging as an authenticated user

Once you are registered in the web service with a username and password, in order to use the same credentials also in PyGMQL you have to use the `pygmql_login` tool. This tool is automatically installed when the library is downloaded and installed (both from github or pip).

On linux/MacOS:

```
pygmql.sh --login
```

On Windows:

```
pygmql_win --login
```

Once the tool is executed the following information will be asked:

- The http *address* of the remote service you want to access
- *Username*
- *Password*

8.4 Library modes

The library mode can be setted in the following way:

```
gl.set_mode("remote") # remote processing of the following operations
gl.set_mode("local")  # local processing of the following operations
```

Notice that the `set_mode()` will act only on the following `materialize()` operations while the previous ones will be performed with the previous setted modality.

The default mode of PyGMQL is local.

8.4.1 The remote execution mode

When the user sets the remote mode and calls the `materialize()` operation, the following actions will be performed

1. The local datasets that are used are uploaded to the remote service. Nothing is done to the remote datasets used in the query (if present) since they are already on the server.
2. A compressed representation of the query is sent to the remote service, decoded and executed
3. Once the execution is complete, the results are downloaded, stored and loaded into a `GDataframe()`.

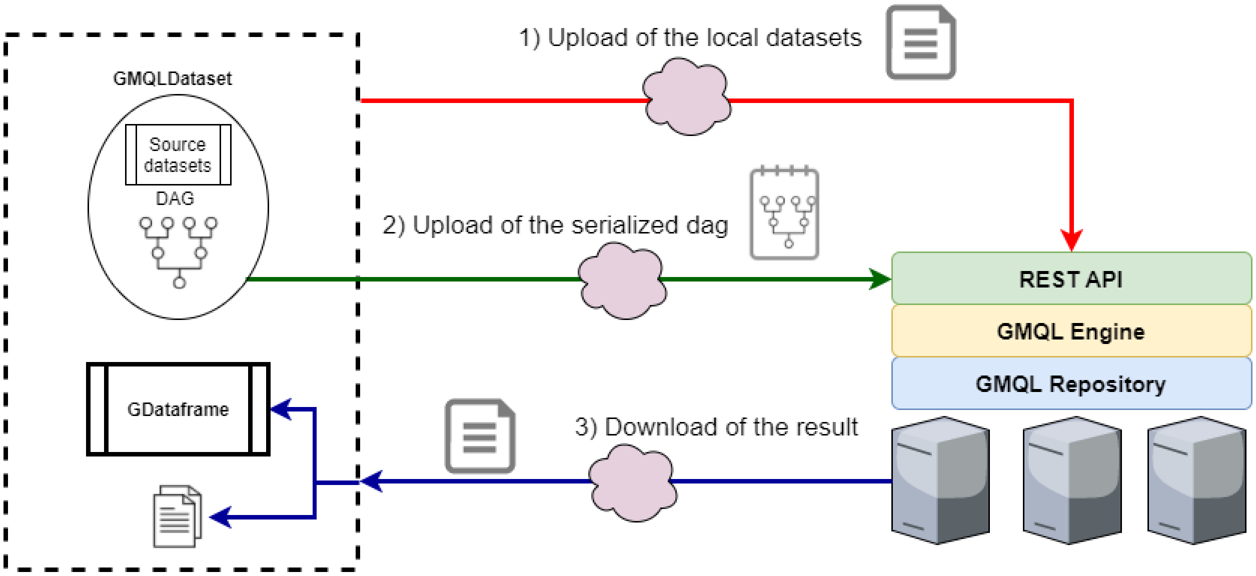
1) Upload of the local datasets
GMQLDataset
Source datasets
DAG
2) Upload of the serialized dag
REST API
GMQL Engine
GMQL Repository
GDataframe
3) Download of the result

CHAPTER 9

Library settings

The following functions define the behavior of the library

9.1 Logging and progress bars

set_progress(*how*)

Enables or disables the progress bars for the loading, writing and downloading of datasets

Parameters **how** – True if you want the progress bar, False otherwise

Returns None

Example:

```
import gmql as gl

gl.set_progress(True)    # abilitates progress bars
# ....do something...
gl.set_progress(False)   # removes progress bars
# ....do something...
```

9.2 Execution Mode

set_mode(*how*)

Sets the behavior of the API

Parameters **how** – if 'remote' all the execution is performed on the remote server; if 'local' all it is executed locally. Default = 'local'

Returns None

9.3 Master Configuration

set_master(*master: str*)
Set the master of the PyGMQL instance. It accepts any master configuration available in Spark.

Parameters **master** – master configuration

Returns None

get_configuration()
Returns the configurations of the current PyGMQL instance

Returns a Configuration object

set_spark_configs(*d*)
Set Spark configurations to be used during the spark-submit. Works only when the master is different from local.

Parameters **d** – a dictionary of {key: values}

Returns None

set_local_java_options(*options: list*)
When the mode is set to local, this function can be used to add JVM specific options before starting the backend. It accepts any Java option.

Parameters **options** – list of string, one for each Java option

Returns None

9.4 Remote Management

get_remote_manager()
Returns the current remote manager

Returns a RemoteManager

login()
Enables the user to login to the remote GMQL service. If both username and password are None, the user will be connected as guest.

logout()
The user can use this command to logout from the remote service

Returns None

set_remote_address(*address*)
Enables the user to set the address of the GMQL remote service

Parameters **address** – a string representing the URL of GMQL remote service

Returns None

CHAPTER 10

Spark and system configurations

The configuration of the Java properties and the Spark environment can be done by getting the singleton instance of the configuration class as follows:

```
conf = gl.get_configuration()
```

Follows the description of this object:

class Configuration

Class containing all the information regarding the system environment and the Spark environment

set_app_name(*name*)

Sets the name of the application in spark, By default it is called "gmql_api"

Parameters **name** – string

Returns None

set_master(*master*)

Set the master of the spark cluster By default it is "local[*]"

Parameters **master** – string

Returns None

set_spark_conf(*key=None*, *value=None*, *d=None*)

Sets a spark property as a ('key', 'value') pair of using a dictionary {'key': 'value', ...}

Parameters

- **key** – string
- **value** – string
- **d** – dictionary

Returns None

set_system_conf(*key=None*, *value=None*, *d=None*)

Sets a java system property as a ('key', 'value') pair of using a dictionary {'key': 'value', ...}

Parameters

- **`key`** – string
- **`value`** – string
- **`d`** – dictionary

Returns None

- genindex
- modindex
- search

CHAPTER 11

Tutorials

11.1 Tutorial 1: Simple example of local processing

11.2 Tutorial 2: Mixing local and remote processing

11.3 Tutorial 3: GWAS on a cloud

CHAPTER 12

Data structures and functions

12.1 Dataset structures

GMQLDataset.GMQLDataset	The main abstraction of the library.
GDataframe.GDataframe	Class holding the result of a materialization of a GMQL-Dataset.

12.2 Dataset loading functions

load_from_path	Loads the data from a local path into a GMQLDataset.
load_from_remote	Loads the data from a remote repository.

12.3 Parsing

For the list of the available parsers go to:

12.3.1 Parsers

Predefined parsers

class BedParser
Standard Full BED Parser of 10 Columns

class ANNParser
Annotation Parser, 6 columns

class BasicParser
Parser for Chr, Start, Stop only (no Strand)

class NarrowPeakParser
Narrow Peaks Parser. 10 columns

class RnaSeqParser
Standard Full BED Parser of 10 Columns

class BedScoreParser
Standard Full BED Parser of 10 Columns

Customizable parser

All the parsers in PyGMQL extend the *RegionParser*

class RegionParser(*gmql_parser=None, chrPos=None, startPos=None, stopPos=None, strandPos=None, otherPos=None, delimiter='t', coordinate_system='0-based', schema_format='del', parser_name='parser'*)
Creates a custom region dataset

Parameters

- **chrPos** – position of the chromosome column
- **startPos** – position of the start column
- **stopPos** – position of the stop column
- **strandPos** – (optional) position of the strand column. Default is None
- **otherPos** – (optional) list of tuples of the type [(pos, attr_name, typeFun), ...]. Default is None
- **delimiter** – (optional) delimiter of the columns of the file. Default " "
- **coordinate_system** – can be {'0-based', '1-based', 'default'}. Default is '0-based'
- **schema_format** – (optional) type of file. Can be {'tab', 'gtf', 'vcf', 'del'}. Default is 'del'
- **parser_name** – (optional) name of the parser. Default is 'parser'

get_gmql_parser()
Gets the Scala implementation of the parser

Returns a Java Object

static parse_strand(*strand*)
Defines how to parse the strand column

Parameters **strand** – a string representing the strand

Returns the parsed result

parse_regions(*path*)
Given a file path, it loads it into memory as a Pandas dataframe

Parameters **path** – file path

Returns a Pandas Dataframe

get_attributes()
Returns the unordered list of attributes

Returns list of strings

get_ordered_attributes()
Returns the ordered list of attributes

Returns list of strings

get_types()
Returns the unordered list of data types

Returns list of data types

get_name_type_dict()
Returns a dictionary of the type {'column_name': data_type, ...}

Returns dict

get_ordered_types()
Returns the ordered list of data types

Returns list of data types

12.4 Aggregates operators

`COUNT`()	Counts the number of regions in the group.
`SUM`(argument)	Computes the sum of the values of the specified attribute
`MIN`(argument)	Gets the minimum value in the aggregation group for the specified attribute
`MAX`(argument)	Gets the maximum value in the aggregation group for the specified attribute
`AVG`(argument)	Gets the average value in the aggregation group for the specified attribute
`BAG`(argument)	Creates space-separated string of attribute values for the specified attribute.
`STD`(argument)	Gets the standard deviation of the aggregation group for the specified attribute
`MEDIAN`(argument)	Gets the median value of the aggregation group for the specified attribute
`Q1`(argument)	Gets the first quartile for the specified attribute
`Q2`(argument)	Gets the second quartile for the specified attribute
`Q3`(argument)	Gets the third quartile for the specified attribute

12.5 Genometric predicates

`MD`(number)	Denotes the minimum distance clause, which selects the first K regions of an experiment sample at minimal distance from an anchor region of an anchor dataset sample.
`DLE`(limit)	Denotes the less-equal distance clause, which selects all the regions of the experiment such that their distance from the anchor region is less than, or equal to, N bases.
`DL`(limit)	Less than distance clause, which selects all the regions of the experiment such that their distance from the anchor region is less than N bases

Continued on next page

Table 4 – continued from previous page

`DGE`(limit)	Greater-equal distance clause, which selects all the regions of the experiment such that their distance from the anchor region is greater than, or equal to, N bases
`DG`(limit)	Greater than distance clause, which selects all the regions of the experiment such that their distance from the anchor region is greater than N bases
`UP`()	Upstream.
`DOWN`()	Downstream.

12.6 Mathematical operators

`SQRT`(argument)	Computes the square matrix of the argument

Python Module Index

g

gmql.dataset.loaders.Loader, 24

Index

A

ANNParser (*class in gmql.dataset.parsers.Parsers*), 39

B

BasicParser (*class in gmql.dataset.parsers.Parsers*), 39
BedParser (*class in gmql.dataset.parsers.Parsers*), 39
BedScoreParser (*class in gmql.dataset.parsers.Parsers*), 40

C

Configuration (*class in gmql.configuration*), 35
cover() (*GMQLDataset method*), 17

D

difference() (*GMQLDataset method*), 21

E

extend() (*GMQLDataset method*), 17

F

flat_cover() (*GMQLDataset method*), 18

G

GDataframe (*class in gmql.dataset.GDataframe*), 27
get_attributes() (*RegionParser method*), 40
get_configuration() (*in module gmql.settings*), 34
get_gmql_parser() (*RegionParser method*), 40
get_name_type_dict() (*RegionParser method*), 41
get_ordered_attributes() (*RegionParser method*), 40
get_ordered_types() (*RegionParser method*), 41
get_reg_attributes() (*GMQLDataset method*), 13
get_remote_manager() (*in module gmql.managers*), 34
get_types() (*RegionParser method*), 41
gmql.dataset.loaders.Loader (*module*), 24
GMQLDataset (*class in gmql.dataset.GMQLDataset*), 13
group() (*GMQLDataset method*), 23

H

head() (*GMQLDataset method*), 24
histogram_cover() (*GMQLDataset method*), 18

J

join() (*GMQLDataset method*), 19

L

load_from_file() (*in module gmql.dataset.loaders.Loader*), 24
load_from_path() (*in module gmql.dataset.loaders.Loader*), 24
load_from_remote() (*in module gmql.dataset.loaders.Loader*), 24
login() (*in module gmql.managers*), 34
logout() (*in module gmql.managers*), 34

M

map() (*GMQLDataset method*), 20
materialize() (*GMQLDataset method*), 23
merge() (*GMQLDataset method*), 22
meta_group() (*GMQLDataset method*), 23
meta_project() (*GMQLDataset method*), 16
meta_select() (*GMQLDataset method*), 14
MetaField() (*GMQLDataset method*), 13

N

NarrowPeakParser (*class in gmql.dataset.parsers.Parsers*), 39
normal_cover() (*GMQLDataset method*), 18

O

order() (*GMQLDataset method*), 20

P

parse_regions() (*RegionParser method*), 40

parse_strand() (*RegionParser static method*), 40
project() (*GMQLDataset method*), 16
project_meta() (*GDataframe method*), 27

R

reg_project() (*GMQLDataset method*), 17
reg_select() (*GMQLDataset method*), 15
RegField() (*GMQLDataset method*), 13
RegionParser (*class in gmql.dataset.parsers.RegionParser*), 40
regs_group() (*GMQLDataset method*), 23
RnaSeqParser (*class in gmql.dataset.parsers.Parsers*), 40

S

select() (*GMQLDataset method*), 14
set_app_name() (*Configuration method*), 35
set_local_java_options() (*in module gmql.settings*), 34
set_master() (*Configuration method*), 35
set_master() (*in module gmql.settings*), 34
set_mode() (*in module gmql.settings*), 33
set_progress() (*in module gmql.settings*), 33
set_remote_address() (*in module gmql.settings*), 34
set_spark_conf() (*Configuration method*), 35
set_spark_configs() (*in module gmql.settings*), 34
set_system_conf() (*Configuration method*), 35
summit_cover() (*GMQLDataset method*), 18

T

to_dataset_files() (*GDataframe method*), 27
to_GMQLDataset() (*GDataframe method*), 27
to_matrix() (*GDataframe method*), 27

U

union() (*GMQLDataset method*), 22
