# Supplementary material for: PyGMQL: scalable data extraction and analysis for heterogeneous genomic datasets
Source: BMC Bioinformatics. 2019 Nov 8;20:560. doi: 10.1186/s12859-019-3159-9 (PMC6842186; doi:10.1186/s12859-019-3159-9)
Supplement: Supplementary file 2 — Additional file 2 Library documentation. Detailed documentation of the software package (PDF). [file 12859_2019_3159_MOESM2_ESM.pdf]

PyGMQL: data extraction and analysis framework for scalable genomic pipelines

Supplementary Material Guide

In this document we describe the supplementary files provided with the manuscript *PyGMQL: data extraction and analysis framework for scalable genomic pipelines*.

Library documentation

The full documentation of the library can be accessed at https://pygmql.readthedocs.io. For completeness we also provide the documentation in PDF format in the file *docs.pdf*.

GitHub repository

The project is hosted at the following GitHub repository: https://github.com/DEIB-GECO/PyGMQL

In this repository are also stored all the applicative examples (https://github.com/DEIB-GECO/PyGMQL/tree/master/examples) and the installation guide for the software (https://github.com/DEIB-GECO/PyGMQL/blob/master/README.md).

Applicative examples

In the main paper we present three different applications of the proposed framework. The relative code (sometimes in an extended form) is provided also as supplementary material at https://github.com/DEIB-GECO/PyGMQL/tree/master/examples in the form of Jupyter Notebook or Python script.

Folder structure

The files are available in the *examples* folder of the project GitHub repository:

```
examples/
    notebooks/
        01_A_simple_example.ipynb
        02a_Mixing_Local_Remote_Processing_SIMPLE.ipynb
        02b_Mixing_Local_Remote_Processing_COMPLETE.ipynb
        03a_GWAS_Local.ipynb
        03b_GWAS_HDFS.ipynb
        03c_GWAS_Google_Cloud.ipynb
        html/
    scripts/
        TICA_gm12878.py
        TICA_hepg2.py
        TICA_k562.py
    data/
        genes/
```

```
HG19_ENCODE_BROAD/
```

In the `examples/notebooks/html` folder can be found the HTML version of the previous notebooks for easy consultation.

Description of the files

Follows a brief description of every file:

`01_A_simple_example.ipynb`

> This is an introductory example which shows a very simple query run on two example dataset which are provided directly with the library. The computation is all performed on the user machine.

`02a_Mixing_Local_Remote_Processing_SIMPLE.ipynb`

> This notebook contains the exact same query shown in the **first example** of the paper. It shows the interaction between the local user machine and a remote system hosting the GMQL repository. In order to successfully run this query, the user must be connected to the internet.

`02b_Mixing_Local_Remote_Processing_COMPLETE.ipynb`

> This notebook contains an extended version of the query shown in the **first example** of the paper, with more data visualization and additional post-processing on the result of the PyGMQL query.

`03a_GWAS_Local.ipynb`

> This notebook contains the same query shown as **second example** in the paper. The only difference with the code shown in the manuscript is that this query is not executed on the Google Cloud File System but in the user local machine. This is provided to enable the execution of this example also to users which do not have access to a Hadoop Cluster or Google Cloud infrastructure.

`03b_GWAS_HDFS.ipynb`

> This notebook contains the code for executing the **second example** of the paper on HDFS. It assumes that the user has loaded the data on Hadoop and has write access to it.

`03c_GWAS_Google_Cloud.ipynb`

> This notebook contains the code for executing the **second example** of the paper on Google Cloud Platform. It assumes that the data has been loaded on Google Cloud Storage.

`TICA.py`

> These three python scripts contain the code to execute the **third example** of the paper. It assumes that the necessary data have been loaded in HDFS.

Data availability

In the `examples/data` folder are present two datasets which are used in the examples 02a, 02b and 03a. For the queries 03b, 03c and TICA_* the user must download the ENCODE BroadPeak, ENCODE NarrowPeak and GENCODE annotation dataset in the GDM format. They can be downloaded from the following link:

https://s3.us-east-2.amazonaws.com/geco-repository/geco-repository-minimal.tar.gz

This tar.gz file contains the following datasets:

- HG19_ENCODE_BROAD: used in query 03b, 03c and TICA_*
- HG19_BED_ANNOTATION: used in query TICA_*

- HG19_ENCODE_NARROW: used in query TICA_*
- HG19_ANNOTATION_GENCODE: used in query TICA_*

Docker image

We provide a docker image that contains the previous applicative examples inside to be run.

NB: it is possible to run in the docker image only the following examples

`01_A_simple_example.ipynb`

`02a_Mixing_Local_Remote_Processing_SIMPLE.ipynb`

`02b_Mixing_Local_Remote_Processing_COMPLETE.ipynb`

`03a_GWAS_Local.ipynb`

This is due to the lack of a proper support for Hadoop on Docker. Therefore, the queries which need the support of an HDFS cluster cannot be executed in the Docker image. In any case, all the examples are provided inside the container.

Installing the Docker image

If you want to run some of the examples provided in the example folder you can directly install the PyGMQL docker image.

```
docker pull gecopolimi/pygmql
```

You can run the docker instance using the following command:

```
docker run --rm \
           --name pygmql_instance \
           -p <port>:8888 \
           gecopolimi/pygmql
```

where you can set `<port>` to any free port number on your machine. This will start a Jupyter Lab server which will run at the address

```
https://localhost:<port>
```

Inside the docker you will find the `example` folder containing both notebooks and scripts.

Note about executing workflows on Hadoop or Google Cloud

In order to run the programs making use of a Spark cluster with an Hadoop file system it is necessary to have:

- A correctly installed Hadoop file system: you can download Hadoop from this link and then follow this guide to setup yours
- A correctly installed Spark distribution: you can download it from this link and then follow the instructions at this link
- The GMQL repository data used in the workflows
    - you can download the whole set of GDM datasets used in the queries from this link
    - unpack the tar.gz file

- use `hdfs dfs put ./geco-repository hdfs:///` to put the contents of the uncompressed folder in HDFS

AWS EMR cluster configurations

We evaluated the performance of the system using Amazon Web Services Elastic Map Reduce, which offers the possibility to specify the Hadoop cluster configuration, the number of nodes and the various instances specifications. For completeness we provide as supplementary material also the AWS command line scripts to setup an EMR cluster for every configuration defined in the paper.

`examples/cluster_configurations/`

`AWS_EMR_1m_1s.sh:` 1 master and 1 slave

`AWS_EMR_1m_3s.sh:` 1 master and 3 slaves

`AWS_EMR_1m_5s.sh:` 1 master and 5 slaves

`AWS_EMR_1m_10s.sh:` 1 master and 10 slaves
